# Reactive oxygen species metabolism and photosynthetic performance in leaves of *Hordeum vulgare* plants co-infested with *Heterodera filipjevi* and *Aceria tosichella*

**DOI:** 10.1007/s00299-020-02600-5

**Published:** 2020-09-21

**Authors:** Mateusz Labudda, Krzysztof Tokarz, Barbara Tokarz, Ewa Muszyńska, Marta Gietler, Mirosława Górecka, Elżbieta Różańska, Anna Rybarczyk-Płońska, Justyna Fidler, Beata Prabucka, Abdelfattah A. Dababat, Mariusz Lewandowski

**Affiliations:** 1 Department of Biochemistry and Microbiology, Institute of Biology, Warsaw University of Life Sciences‐SGGW, Nowoursynowska 159, 02-776 Warsaw, Poland; 2 Department of Botany, Physiology and Plant Protection, Faculty of Biotechnology and Horticulture, University of Agriculture in Krakow, Krakow, Poland; 3 Department of Botany, Institute of Biology, Warsaw University of Life Sciences‐SGGW, Warsaw, Poland; 4 International Maize and Wheat Improvement Center (CIMMYT), Soil Borne Pathogens Program, Ankara, Turkey; 5 Department of Plant Protection, Section of Applied Entomology, Institute of Horticultural Sciences, Warsaw University of Life Sciences‐SGGW, Warsaw, Poland

**Keywords:** Barley, Cyst nematode, Double pest infestation, Oxidative stress, Photosynthetic apparatus, Wheat curl mite

## Abstract

**Key message:**

Defence responses of cyst nematode and/or wheat curl mite infested barley engage the altered reactive oxygen species production, antioxidant machinery, carbon dioxide assimilation and photosynthesis efficiency.

**Abstract:**

The primary aim of this study was to determine how barley responds to two pests infesting separately or at once; thus barley was inoculated with *Heterodera filipjevi* (Madzhidov) Stelter (cereal cyst nematode; CCN) and *Aceria tosichella* Keifer (wheat curl mite; WCM). To verify hypothesis about the involvement of redox metabolism and photosynthesis in barley defence responses, biochemical, photosynthesis efficiency and chlorophyll *a* fluorescence measurements as well as transmission electron microscopy were implemented. Inoculation with WCM (apart from or with CCN) brought about a significant suppression in the efficiency of electron transport outside photosystem II reaction centres. This limitation was an effect of diminished pool of rapidly reducing plastoquinone and decreased total electron carriers. Infestation with WCM (apart from or with CCN) also significantly restricted the electron transport on the photosystem I acceptor side, therefore produced reactive oxygen species oxidized lipids in cells of WCM and double infested plants and proteins in cells of WCM-infested plants. The level of hydrogen peroxide was significantly decreased in double infested plants because of glutathione–ascorbate cycle involvement. The inhibition of nitrosoglutathione reductase promoted the accumulation of *S*-nitrosoglutathione increasing antioxidant capacity in cells of double infested plants. Moreover, enhanced arginase activity in WCM-infested plants could stimulate synthesis of polyamines participating in plant antioxidant response. Infestation with WCM (apart from or with CCN) significantly reduced the efficiency of carbon dioxide assimilation by barley leaves, whereas infection only with CCN expanded photosynthesis efficiency. These were accompanied with the ultrastructural changes in chloroplasts during CCN and WCM infestation.

## Introduction

The changing climatic conditions, for example long, warm spring, hot summer, lack of the frost during winter and changed rainfall patterns in the temperate climate zone, are conducive to pests and pathogens gradation on crop plants. All these circumstances can contribute to a decrease in crop productivity (Gregory et al. 2009). Cyst nematodes and eriophyoid mites are significant pests affecting cereals. The cereal cyst nematode (CCN) *Heterodera filipjevi* (Madzhidov) Stelter (Nematoda: Heteroderidae), is one of the global scale biotrophic phytopathogen of cereals (Toumi et al. 2018). Upon the root infestation, infective larvae of CCN induce syncytia in the vascular cylinder. These multicellular structures are becoming the solely sources of nutrients drawing from the host plant. What is important, the parasitism of *H. filipjevi* specimens on roots of the colonised cereal plants may cause even to 50% grain yield losses (Pariyar et al. 2016; Dababat and Fourie 2018). The wheat curl mite (WCM), *Aceria tosichella* Keifer (Acariformes: Eriophyoidea), is around the world scattered eriophyoid pest of cereal plants (Kuczyński et al. 2016). WCM is a serious problem on cereals, including barley, because it damages leaves, what can impair overall physiology of plant, but until now there have been very little published reports regarding biochemical/physiological interactions with cereal hosts (Skoracka et al. 2018b). Furthermore, WCM can effectively spread plant pathogenic viruses such as the wheat streak mosaic virus (WSMV) from the family Potyviridae. This is the most significant economic impact of WCM on cereal yielding. As a result of WCM feeding or WSMV transmission on cereal leaves, the grain yield losses may reach up to 40% (Skoracka et al. 2017, 2018a; Aguirre-Rojas et al. 2019).

It is well known that in plant organisms suffering from the pathogen or pest infestation, the altered metabolism of reactive oxygen species (ROS) occurs (Labudda et al. 2018; Woźniak et al. 2019). ROS are continuously produced in various metabolic pathways, including photosynthesis. Besides, the ROS metabolism and the regulation of photosynthesis are tightly linked (Foyer 2018). The chloroplastic ROS have origins in photosystem I (PSI) and photosystem II (PSII) and they are produced when the absorption of light quanta outbalances the efficiency of photosynthesis and the photoprotection responses are interrupted (Khorobrykh et al. 2020). ROS have a dual role in plant physiology. On the one hand, they participate in various developmental processes under normal plant growth and act as signalling molecules in acclimatization to environmental stresses. On the other hand, the exaggerated ROS level in plant cells can lead to the oxidation of proteins, lipids, nucleic acids, saccharides and pigments (Muszyńska and Labudda 2019; Muszyńska et al. 2019). To render the pauperization of plant cell functions impossible, the ROS content in cells is mastered by enzymatic and non‐enzymatic antioxidant mechanisms. The enzymatic machinery consists of superoxide dismutase (SOD), catalase (CAT), multifarious peroxidases and enzymes of the Foyer–Halliwell–Asada cycle (Labudda and Azam 2014; Muszyńska et al. 2018; Kapoor et al. 2019). The non‐enzymatic mechanisms are mainly based on the reduced glutathione (GSH) and ascorbate (ASA) and an abundant group of the phenol metabolites (Saxena et al. 2016; Durak et al. 2019; Muszyńska et al. 2020). Among the phenols, salicylic acid (SA) is a phytohormonal molecule, which modulates many plant reactions to the environmental stresses (Morkunas et al. 2011, 2018; Maruri-López et al. 2019; Formela-Luboińska et al. 2020).

While the knowledge about the physiological basis of plant responses to the infection by one pathogen or pest species is constantly increasing, the number of research reports attempting to explain the complex mechanisms of double biotic stress is extremely limited. In our research team, we have investigated the physiological response of plants to the cyst nematode infection for several years (Labudda et al. 2016a, b, 2018, 2020a, b; Labudda 2018). In addition, even though WCM is a very intensively studied mite on a global scale, little is known about the responses of host plants to WCM infection (Skoracka et al. 2018b). Our goal was to find out if a pest infection induces in barley leaves various but interconnected defence responses at different levels of plant organization (from biochemical, through physiological, to ultrastructural). We hypothesized that these responses against biotic stress factors could fit the concept of ‘fan-shaped’ defence response engaging a holistic and complex but integrated plant stress response. This ‘fan-shaped’ type of plant defence response was originally proposed by Sanità di Toppi and Gabbrielli (1999) for abiotic stress (cadmium exposure). However, so far such a hypothesis has not been considered in the literature in the context of cereal plant response to the stress caused by the infestation by two pests. Therefore, we undertook to examine parameters of two primeval stress-sensitive metabolic areas, namely the redox balance and photosynthetic efficiency (Ślesak et al. 2019). The research approach used by us seems to be particularly interesting because CCN attacks roots, while WCM infests leaves, what should cause consequences in the holistic plant response to biotic stressors. To learn about these, we implemented biochemical and enzymological methods, photosynthesis efficiency and chlorophyll *a* fluorescence measurements accompanied by observations under a transmission electron microscope.

## Materials and methods

### Plants

The spring barley *Hordeum vulgare* L. cv. ‘Airway’ seeds were washed in tap water for two hours. Then, they were surface decontaminated in 5% sodium hypochlorite with 0.2% polysorbate 20 as a surfactant for 10 min with stirring. Next, they were rinsed under tap water for 1 hour and an incubation, for 1 hour in 0.2% Plant Preservative Mixture (PPM) (Plant Cell Technologies, Inc., Washington DC, USA) to eliminate potential microbial contamination, was performed. The decontaminated seeds (embryos upwards) were put into Petri dishes (9 cm diameter) on a 0.2% PPM‐soaked filter paper and covered. After 18 hours incubation in the fridge at 4 °C in the dark, the seeds were kept in the dark at 23 °C for 2 days (Labudda et al. 2020b). Twelve germinated seeds were subsequently planted into a black plastic pot (25 × 25/26 cm) with saucer. Pot was filled with a commercial horticultural substrate consisted of mixed low-moor and high-moor peats (0–20 mm fraction). Substrate had no addition of mineral fertilizers, its pH in water was in the range of 5.6–6.8 and before planting it was autoclaved at 121 °C, 0.1 MPa for 20 min. Aliquot of 150 ml of sterile 0.2 × Knop medium (pH 6.4) was added to the pots. Plants were cultivated in a growth chamber MLR‐350 (Sanyo, Tokyo, Japan) at 25 °C during the day and at 23 °C at night with a 16 h/8 h day/night cycle under a photosynthetic photon flux density of 100 ± 25 μmol m^−2^ s^−1^ and at 50% humidity. Every 2 days, plants were watered with 100 ml of water.

### Animals

The stock colony of *Aceria tosichella* Keifer was maintained for 45 generations on *H. vulgare* plants growing in pots in laboratory of Department of Plant Protection (Section of Applied Entomology) at Warsaw University of Life Sciences‐SGGW. *A. tosichella* biotype MT-1 (GenBank: JF920077) was used (Skoracka et al. 2013). WCM-infested plants were cultivated in a growth chamber MLR‐350 at 27 °C during the day and at 25 °C at night with a 16 h/8 h day/night cycle under a photosynthetic photon flux density of 100 ± 25 μmol m^−2^ s^−1^ and at 50% humidity. Each pot was put in rearing cages consisting of metal frames tightly covered with nylon mesh bags.

The *Heterodera filipjevi* (Madzhidov) Stelter cysts were collected from naturally cyst nematode‐infested experimental *Triticum aestivum* fields of the International Maize and Wheat Improvement Center (CIMMYT) in Yozgat (39° 08′ N, 34° 10′ E; altitude 985 m a.s.l.) in the Central Anatolian Plateau of Turkey. Cysts were extracted from rhizospheres and roots of *Triticum aestivum* plants harvested at the end of the growing season. The modified extraction protocol was implemented (Ashrafi et al. 2017). Hatching of *H. filipjevi* pre-parasitic juveniles was provoked by the incubation of cysts in sterile 0.003 M ZnCl_2_ at 17 °C. Freshly hatched pre-parasitic juveniles were washed six times and then suspended in sterile water.

### Plant inoculation with mites and nematodes

Pots with 7-day‐old plants were divided into four groups: nematode-uninoculated and WCM-uninoculated controls (C), CCN-inoculated (N), WCM-inoculated (WCM) and both CCN-inoculated and WCM-inoculated (N + WCM) plants. Roots of plants from the N and N + WCM groups were inoculated with approximately 2400 freshly hatched pre-parasitic *H*. *filipjevi* juveniles (per pot) and plants were watered with 100 ml of water. Leaves of the plants from the WCM and N + WCM groups were inoculated with three females (per plant) of WCM adapted to feed on barley and plants were watered with 100 ml of water. Control plants were watered as well. Each pot was put in rearing cages consisting of metal frames tightly covered with nylon mesh bags. Control and infested plants were sampled after 18 days of experiment. This sampling time point was chosen based on our previous published observations (Labudda et al. 2020b) reflected the dynamics of growth and development of *H*. *filipjevi* larvae in spring barley (the same cultivar as tested in this article) roots under conditions of pot experiment. After 14 days post‐inoculation (dpi) but before 21 dpi, most sedentary J2 larvae moulted to J3 larvae, which indicated that the 18-dpi syncytia met their nutritional demands. About 18 dpi, the J3 larvae fed intensively to reach the J4 stage and sexual maturity after 21 dpi and start reproducing. The sampling time at 18 dpi was also rational and justified by the fact that at this time the barley plants showed clear signs of being infected by the WCM (leaf curl and yellowing) but the WCM population had not yet developed to the point that could cause the death of infested plants. For biochemical measurements, the leaf bulked samples were collected, and experiments were conducted in three biological replicates.

### Biochemical measurements

#### Superoxide anion and hydrogen peroxide (H_2_O_2_)

Assay of superoxide anions level was conducted according to Doke’s method (Mai et al. 2013). Leaves (100 mg) were immersed in millilitre of the 0.01 M K/Na phosphate buffer (pH 7.8) containing 0.05% nitro blue tetrazolium (NBT) and 0.01 M NaN_3_. Samples were kept in the dark for an hour at room temperature (RT). Next, reaction mixtures (without plant material) were incubated at 85 °C for 15 min and forthwith cooled on ice. The superoxide anions level was expressed as absorbance at 580 nm per gram of fresh leaf weight (FW).

The H_2_O_2_ amount was measured according to the method by Junglee et al. (2014). Leaves (100 mg) were disintegrated in medium containing 250 µl of 0.01 M K/Na-phosphate buffer (pH 5.8), 250 µl of 0.1% trichloroacetic acid and 500 µl of 1 M KI. Supernatants, collected after homogenate centrifugation (4 °C, 15 min, 16,000×*g*), were incubated in the dark for 20 min at RT. Next, the samples were centrifuged (10 min, 16,000×*g*), and the absorbance was read at 350 nm in Nunc U-bottom 96-well plate (Thermo Scientific, Waltham, MA, USA) on a Varioskan LUX Multimode Microplate Reader (Thermo Scientific, Waltham, MA, USA). The hydrogen peroxide amount was calculated from a standard curve and expressed per gram of FW.

#### Enzymatic parameters

Leaves (200 mg) were crushed in a mortar with quartz sand and 2 ml of ice-cold buffered mixture (pH 7.2) containing 0.05 M tris(hydroxymethyl)aminomethane hydrochloride (Tris)-HCl, 0.002 M 2-mercaptoethanol, 0.001 M ethylenediaminetetraacetic acid (EDTA), 5% propane-1,2,3-triol, 0.001 M phenylmethylsulfonyl fluoride, 0.005 M magnesium chloride, 2% polyvidone. Enzymatic extracts were obtained by the centrifugation of homogenates (4 °C, 20 min, 16,000×*g*).

The activity of superoxide dismutase (SOD) was estimated by Kostyuk and Potapovich (1989) method. An assay mixture was made by mixing equal aliquots of 0.025 M EDTA and 0.067 M K/Na phosphate buffer (pH 7.8). The pH value of assay mixture was precisely adjusted to 10 with *N*,*N*,*N*′,*N*′-tetramethylethane-1,2-diamine. Next, 50 µl of assay mixture and one-hundred 20 µl distilled water were pipetted to 5 µl of enzymatic extract. The enzymatic reaction was initiated by the addition of 5 µl of 2.5 × 10^–6^ M 2-(3,4-dihydroxyphenyl)-3,5,7-trihydroxy-4*H*-chromen-4-one suspended in (CH_3_)_2_SO. Assays were made in Nunc U-bottom 96-well plate on a Varioskan LUX Multimode Microplate Reader. The absorbance at 406 nm was recorded for 20 min with reads every minute. The arbitrary unit of SOD activity was ascertained as 0.01 decrease of absorbance after minute per gram of FW.

The activity of catalase (CAT) was estimated by Aebi (1984) method. The enzymatic extract (2 µl) was mixed with 18 µl of 0.05 M Tris–HCl buffer (pH 7.2) and 10 µl of 0.168% H_2_O_2_ in the same buffer. Assays were made at 37 °C in UV-Star 96-well plate (Greiner, Monroe, NC, USA) on a Varioskan LUX Multimode Microplate Reader. The absorbance at 240 nm was monitored for 10 min with reads every minute. The CAT activity was expressed as a breakdown of micromoles of H_2_O_2_ per minute and gram of FW.

The peroxidase activity (POD) was estimated by Lück (1962) method. The enzymatic extract (5 µl) was mixed with a reagent consisting of 0.49% p-phenylenediamine and 0.049% H_2_O_2_ in 0.05 M Tris–HCl buffer, pH 7.2 or 8.8. Assays were conducted at 37 °C in Nunc U-bottom 96-well plate on a Varioskan LUX Multimode Microplate Reader. The absorbance at 485 nm was read for 10 min with reads every minute. The POD activity was expressed in arbitrary unit, separately for pH 7.2 (POD_7.2_) and 8.8 (POD_8.8_). The unit of POD activity was defined as 0.1 increase of absorbance after minute per gram of FW.

The guaiacol peroxidase activity (GOPX) was estimated by Chance and Maehly (1955) method. The enzymatic extract (5 µl) was mixed with a reagent consisting of 0.005 M guaiacol and 0.0025 M H_2_O_2_ in 0.05 M acetic buffer, pH 5.6. Assays were performed at 37 °C in Nunc U-bottom 96-well plate on a Varioskan LUX Multimode Microplate Reader. The absorbance at 470 nm was measured for 10 min with reads every minute. The GOPX activity was expressed in micromoles of produced tetraguaiacol (*ɛ* = 26.6 mM^−1^ cm^−1^) per minute and gram of FW.

The ascorbate peroxidase activity (APX) was estimated by Nakano and Asada (1981) method. The enzymatic extract (5 µl) was mixed with a reagent consisting of 0.05 M Tris–HCl buffer, pH 7.2, 0.002 M ASA, 0.005 M EDTA and 0.0001 M H_2_O_2_. The APX activity was measured at 25 °C in UV-Star 96-well plate on a Varioskan LUX Multimode Microplate Reader by following the rate of ASA oxidation for 10 min with absorbance reads every minute at 290 nm. The APX activity was expressed in micromoles of ASA decomposition (ɛ = 2.8 mM^−1^ cm^−1^) per minute and gram of FW.

The dehydroascorbate reductase (DHAR) activity was estimated using Trümper et al. (1994) method. The enzymatic extract (10 µl) was mixed with a reagent consisting of 0.05 M Tris–HCl buffer, pH 7.2, 0.004 M GSH and 0.001 M dehydroascorbic acid (DHA). The DHAR activity was measured at 30 °C in UV-Star 96-well plate on a Varioskan LUX Multimode Microplate Reader by following the rate of DHA reduction for 10 min with absorbance reads every minute at 265 nm. The DHAR activity was expressed in micromoles of ASA production (ɛ = 14 mM^−1^ cm^−1^) per minute and gram of FW.

The glutathione reductase (GR) activity was estimated by Foyer and Halliwell (1976) method. The enzymatic extract (10 µl) was mixed with a reagent consisting of 0.05 M Tris–HCl buffer, pH 7.2, 0.00025 M nicotinamide adenine dinucleotide phosphate (NADPH), 0.001 M EDTA and 0.001 M oxidized glutathione (GSSG). Assays were performed at 37 °C in Nunc U-bottom 96-well plate on a Varioskan LUX Multimode Microplate Reader and the change in absorbance at 340 nm was noted for 20 min with reads every minute. The GR activity was expressed as micromoles of oxidized NADPH per minute and gram of FW.

The nitrosoglutathione reductase (GSNOR) activity was estimated by Sakamoto et al. (2002) method. The enzymatic extract (10 µl) was mixed with a reagent consisting of 0.05 M Tris–HCl buffer, pH 7.2, 0.0002 M nicotinamide adenine dinucleotide (NADH), 0.0005 M EDTA and 0.0006 M *S*-nitrosoglutathione (GSNO). Assays were carried out at 37 °C in Nunc U-bottom 96-well plate on a Varioskan LUX Multimode Microplate Reader and change in absorbance at 340 nm was noted for 20 min with reads every minute. The GSNOR activity was expressed as micromoles of oxidized NADH per minute and gram of FW.

The arginase (ARG) activity in enzymatic extract was measured by Labudda et al. (2016a) method. After the activation of ARG (0.005 M manganese dichloride, 56 °C, 10 min), the enzymatic extracts were kept at 37 °C with 0.25 M L‐arginine (pH 9.6). The reactions were terminated after 60 min by adding the reagent consisting of sulphuric acid:orthophosphoric acid:distilled water (1:3:7, *v*/*v*/*v*). Next, 9% α‐isonitrosopropiophenone dissolved in 96% ethanol was added, and samples were incubated at 96 °C for 45 min. Next, samples were placed for 10 min in the dark at RT. The absorbance was recorded at 550 nm in Nunc U-bottom 96-well plate on a Varioskan LUX Multimode Microplate Reader. The content of produced urea was estimated based on the standard curve and the ARG activity was expressed as micromoles of urea per hour and gram of FW.

#### Phenolic metabolites

Leaves (100 mg) were crushed in a mortar with quartz sand on ice-bath. The phenolic metabolites were extracted with 5 ml of ice‐cold 80% methyl alcohol and tissue homogenates were centrifuged for 15 min at 4 °C (16,000×*g*). The amount of total phenols, hydroxycinnamoyl tartaric acid esters, flavonols and anthocyanins was estimated by Fukumoto and Mazza (2000) method. The methyl alcohol extracts were mixed with 0.1% HCl solution prepared in 96% ethanol and 2% HCl solution prepared in distilled water. Then 15 min after incubation in the dark at RT, the absorbance was noted in UV-Star 96-well plate (Greiner) on a Varioskan LUX Multimode Microplate Reader. The absorbance at 280, 320, 360 and 520 nm reflected total phenol, hydroxycinnamoyl tartaric acid ester, flavonol and anthocyanin contents, respectively. The chlorogenic acid (total phenols), caffeic acid (hydroxycinnamoyl tartaric acid esters), quercetin (flavonols) and cyanidin (anthocyanins) were used as equivalents for assessment of phenolic metabolites. To measure the polyphenol content, the Folin-Ciocalteu method was used (Labudda et al. 2016b). Briefly, the methyl alcohol extract was mixed with distilled water and Folin-Ciocalteu reagent (POCH, Gliwice, Poland). Samples were kept at RT for 4 min, and 1 M saturated Na_2_CO_3_ solution was pipetted and incubation at 40 °C for 30 min was carried out. The absorbance was noted at 740 nm in Nunc U-bottom 96-well plate on a Varioskan LUX Multimode Microplate Reader and the polyphenol content was calculated as gallic acid equivalent. The results of the phenolic metabolite levels were expressed in milligrams of the respective equivalents per hundred grams of FW.

The total salicylic acid (free and conjugated forms of SA) contents were measured by the reversed-phase high-performance liquid chromatography (RP-HPLC) with detection of fluorescence according to Szkop et al. (2017) method with minor modifications. Leaves (100 mg) were macerated in a mortar with quartz sand and 0.4 M dipotassium phosphate. Samples were vigorously agitated for 15 min at 70 °C and centrifuged at 16,000×*g* for 10 min. Next, supernatants were mixed with 10 M HCl and the hydrolysis at 95 °C for 90 min was conducted. Subsequently, ethyl ethanoate was added to the hydrolysates and samples were vigorously agitated and centrifuged at 16,000×*g* for 10 min. The organic phases were mixed with phosphate buffer (pH 7.8) and samples were vigorously agitated and centrifuged at 16,000×*g* for 10 min. The aqueous phases were collected, filtered and pipetted into the vials. The HPLC analysis was performed using system consisted of a binary pump (Model 1525, Waters Corporation, Milford, MA, USA), a fluorometric detector (Model 474, Waters Corporation) and an autosampler (Model 717plus, Waters Corporation). Separations were carried out at RT on a C8 column (Symmetry 4.6 × 150 mm, 5 μm, Waters Corporation) guarded by a C8 precolumn (Symmetry 3.9 × 20 mm, 5 μm, Waters Corporation) with a linear gradient elution. The content of SA was calculated based on the external standard curve prepared with the use of HPLC-grade SA (Sigma-Aldrich, Saint Louis, MO, USA).

#### Lipid peroxidation and protein carbonylation

The measurement of the 2-thiobarbituric acid reactive substances (TBARs) content was estimated by Hodges et al. (1999) method. Two hundred microlitres of methyl alcohol extract (obtained as described in Phenolic metabolites paragraph) was pipetted to 800 µl 0.5% 2-thiobarbituric acid prepared in 20% trichloroacetic acid. Samples were incubated at 90 °C for 20 min and reactions were terminated on ice. Samples were centrifuged (16,000×*g*) for 10 min and the absorbance was read at 440, 532 and 600 nm in Nunc U-bottom 96-well plate on a Varioskan LUX Multimode Microplate Reader. TBARs content was counted and expressed in micromoles per gramme of FW.

To estimate protein carbonylation (carbonyl groups, C = O) content, leaves (100 mg) were crushed with liquid nitrogen in mortar. Two millilitres of extraction medium (0.1 M phosphate buffer pH 7.2 with 0.001 M EDTA, and 0.1% Triton X-100) was added into the macerated tissue. Samples were centrifuged (16,000×*g*) for 15 min at 4 °C. The protein content was measured in supernatants with Bradford reagent and bovine serum albumin (Sigma-Aldrich) as the protein standard. Aliquots containing 180 µg of soluble proteins were prepared, and next proteins were precipitated with cold propan-2-one. The protein carbonylation was measured by derivatization of protein carbonyls with 2,4-dinitrophenylhydrazine (DNPH) using Levine et al. (1994) method. Briefly, 0.01 M DNPH in 2.5 M hydrochloric acid was added to proteins and incubated at RT for hour in darkness with mixing every 15 min. Next, the protein pellets were washed three times with cold ethyl alcohol/ethyl ethanoate (1:1) mixture. Dinitrophenyl group (DNP)-labelled proteins were solubilised in 90 µl of rehydration medium containing 7 M carbonic diamide, 2 M thiocarbamide, 4% 3-[(3-cholamidopropyl)dimethylammonio]-1-propanesulfonate and 0.04 M Cleland’s reagent.

Samples containing 5 µg of derivatized proteins were mixed (1:1, *v*/*v*) with a sample buffer containing 0.126 M Tris–HCl (pH 6.8), 20% propane-1,2,3-triol, 4% sodium dodecyl sulphate (SDS), 10% 2-mercaptoethanol and 0.004% bromophenol blue. Denatured protein samples were electrophoresed on 11% SDS acrylamide gel in a 0.025 M Tris, 0.192 M 2-aminoethanoic acid and 0.1% SDS running buffer (pH 8.3) at 60 V for 15 min followed by hour at a constant current of 0.025 A per gel until the blue dye front reached the bottom of the gel (Mini-Protean electrophoresis system; Bio-Rad, Hercules, CA, USA). SDS–polyacrylamide gel electrophoresis separated proteins were transferred to a nitrocellulose membrane (Mini-Protean electrophoresis system; Bio-Rad). After hour of blocking with 5% skimmed milk at RT, the membrane was incubated with anti-DNP polyclonal rabbit antibodies (Sigma-Aldrich; 1:1500) in phosphate-buffered saline (PBS) (pH 7.4) with 0.5% Tween 20. Alkaline phosphatase-conjugated goat antibodies against rabbit IgG (Sigma-Aldrich; 1:20,000) were used as the secondary antibodies. The blots were developed by NBT/5-bromo-4-chloro-3′-indolyphosphate (BCIP) reagent containing 0.015% of BCIP and 0.03% of NBT in 0.1 M Tris–HCl buffer, pH 9.5 supplemented with 0.1 M sodium chloride and 0.05 M magnesium dichloride. The molecular weight (MW) of proteins was determined by SpectraTM Multicolor Broad Range Protein Marker (Thermo Scientific). Blots were digitalized with G:BOX EF2 (Syngene, Cambridge, UK) and the intensity of bands was quantified as % volume with free BioVision software (Vilber, Collégien, France). The average intensity of all bands was ascertained. Results were compared with C plants, to which a value of 100% has been ascribed.

### Photosynthetic measurements

#### Photosynthetic pigments

The photosynthetic pigments were estimated by Lichtenthaler (1987) method. Leaves (100 mg) were homogenised in ice‐cold 80% propan-2-one with addition of calcium carbonate and centrifuged (16,000×*g*) for 15 min at 4 °C. The absorbance of propan-2-one extracts was read at 470, 646 and 663 nm using double-beam spectrophotometer U-2900 (Hitachi High-Technologies Corporation, Tokyo, Japan). The chlorophyll *a* (chl *a*), chlorophyll *b* (chl *b*) and carotenoid (car) contents were calculated according to Wellburn (1994). Total chlorophylls (chl *a* + *b*), the ratio of chlorophyll *a* to *b* (chl *a*:*b*) and the ratio of chl *a* + *b* to car (chl *a* + *b*:car) were also calculated.

#### Photosynthesis efficiency

Actual photosynthesis efficiency was evaluated by measuring gas exchange (H_2_O and CO_2_) and maximal efficiency of photosynthesis light reactions by photosynthetic light response curve measurement using a portable open gas-exchange system (LCpro-SD; ADC BioScientific Ltd., Hoddesdon, UK) equipped with a 6.24 cm^2^ cuvette and a mixed Red/Blue LED Light Source Head. Measurements were carried out on fully developed leaves of five plants from each treatment. In gas exchange measurement, to allow photosynthesis to reach the steady state, each leaf was adapted for 2 min in the cuvette. The measurements were performed in CO_2_ saturated conditions (650 μmol mol^−1^): 300 μmol s^−1^ of airflow, 50–55% relative humidity within the cuvette, 25 °C leaf temperature and under the 175 μmol m^−2^ s^−1^ red/blue light intensity. Net photosynthesis [PN (μmol CO_2_ m^−2^ s^−1^)], stomatal conductance [Gs (mmol H_2_O m^−2^ s^−1^)] and rate of transpiration [E (mmol H_2_O m^−2^ s^−1^)] were evaluated. Photosynthetic light response curves were recorded on other leaf each of the same five plants. Light Source Head was used for a stepwise reduction of photosynthetically active radiation (PAR) ranging from 1500 to 0 μmol (quanta) m^−2^ s^−1^ (in 300, 100, 50, 20, 0, 100, 300, 500, 1000, 1500, 300 and 100 μmol (quanta) m^−2^ s^−1^ steps). The leaves were adapted to each of the light intensities for 5, 5, 3, 2, 5, 5, 5, 5, 5, 5, 5 and 5 min, respectively, before data point recording. Air flow, relative humidity and CO_2_ concentration inside the cuvette were the same as described for gas exchange measurement.

#### Chlorophyll *a* fluorescence

Chlorophyll *a* fluorescence measurement was carried out with Handy-PEA (Hansatech, King’s Lynn, UK) fluorometer using standard procedures. Ten leaves (two on each of five plants) from each treatment were dark-adapted for 25 min. The fluorescence was induced by red light: max = 650 nm, 2000 mol m^−2^ s^−1^. Selected functional and structural photosynthetic parameters were calculated (Jiang et al. 2008; Kalaji et al. 2011) (Table 1). Table 1 was compiled according to Piwowarczyk et al. (2018). Recorded curves were analysed using the fluorometer producer’s softsware (PEA-Plus). Evaluated parameters allow for assessment of photosystem II (PSII) efficiency.

**Table 1:** Directly measured and calculated structural and functional photosynthetic parameters

Abbreviations		Descriptions
*F*_0_	Directly measured parameters	Minimum fluorescence when all photosystem II (PSII) reaction centres (RCs) are open
*F*_*M*_		Maximum fluorescence, when all PSII reaction centres are closed
Area		Total complementary area between fluorescence induction curve and F_M_
*F*_*V*_	Parameters calculated from measured parameters	Variable fluorescence
*F*_*V*_/*F*_*M*_		Maximum quantum yield of PSII
*F*_*V*_/*F*_0_		Efficiency of the oxygen-evolving complex on the donor side of the PSII
*V*_*J*_		Relative variable fluorescence at 2 ms (J-step), that refers to the number of closed RCs relative to the total number of RCs
*V*_*I*_		Relative variable fluorescence at 30 ms (I-step); that reflects the ability of photosystem I (PSI) and its acceptors to oxidize reduced plastoquinone
*S*_*m*_		Normalized total complementary area above the OJIP transient (reflecting multiple-turnover the primary quinone acceptor of PSII (Q_A_) reduction events) or total electron carriers per RC
ABS/RC	Specific energy fluxes per Q_A-_ reducing PSII reaction centre	Absorption flux per RC; that reflects the proportion between chlorophyll *a* molecule amounts in fluorescence-emitting antenna complexes and in the active reaction centres
TR_o_/RC		Trapped energy flux per RC at *t* = 0
ET_o_/RC		Electron transport flux per RC at *t* = 0
DI_o_/RC		Dissipated energy flux per RC at *t* = 0
*φ*_Po_	Quantum yields or flux ratios	Maximum quantum yield of primary photochemistry at *t* = 0; that indicates the probability of trapping the energy of absorbed photons by PSII reaction centres
*φ*_Eo_		Quantum yield for electron transport at *t* = 0*;* that reflects efficiency of electron transfer from Q_A‾_ to electron transport chain beyond
*ψ*_Eo_		Probability (at time 0) that trapped exciton moves an electron into the electron transport chain beyond Q_A_
*ρ*_Ro_		Efficiency with which a trapped exciton can move an electron into the electron transport chain from Q_A‾_ to the PSI and electron acceptors
*δ*_Ro_		Efficiency with which an electron can move from the reduced intersystem electron acceptors to the PSI end electron acceptors
*φ*_Ro_		Quantum yield for the reduction of end acceptors of PSI per photon absorbed
ABS/CS_o_	Phenomenological energy fluxes per excited cross section	Absorption flux per cross-section (CS) at *t* = 0; represents the amount of photon energy absorbed by the antenna associated with active and inactive reaction centres of PSII and their relationship
TR_o_/CS_o_		Trapped energy flux per CS at *t* = 0
ET_o_/CS_o_		Electron transport flux per CS at *t* = 0
DI_o_/CS_o_		Dissipated energy flux per CS at *t* = 0
RC/CS_o_	Density of RC	Amount of active PSII RCs per CS at *t* = 0

Abbreviations and descriptions were adopted according to Jiang et al. (2008), Kalaji et al. (2011), Goltsev et al. (2016) and Piwowarczyk et al. (2018)

### Examination of chloroplast ultrastructure

The leaf blade fragments (about 2 × 2 mm in size) were sampled from the central barley leaf part from each treatment. They were fixed in 2% glutaric dialdehyde and 2% polyoxymethylene dissolved in 0.1 M cacodylate buffer (pH 7.2) for 3 h. After four times rinsing in 0.1 M cacodylate buffer, samples were post-fixed in 2% osmium tetroxide for 2 h, dehydrated in increasing ethyl alcohol concentrations, propylene oxide and finally infiltrated with EPON 812 epoxy resin (Fluka, Buchs, Switzerland). Ultra‐thin (60 nm) sections were taken with a Leica UCT ultramicrotome (Leica Microsystems, Wetzlar, Germany) and stained with uranyl acetate and lead citrate. Sections were examined in an FEI 268D ‘Morgagni’ (FEI Corp., Hillsboro, OR, USA) transmission electron microscope operating at 80 kV and a SIS ‘Morada’ digital camera (Olympus‐SIS, Münster, Germany) was used for acquisition of images.

### Statistical analysis

Representative data were presented as the means ± SD. Results were subjected to one-way analysis of variance (ANOVA). The significant differences between experimental groups were determined using Tukey’s honest significant difference test at *p* < 0.05. Statistical analysis was performed using Statistica program, version 13.3 (TIBCO Software Inc., Palo Alto, CA, USA).

## Results

### ROS

**Fig. 1:**
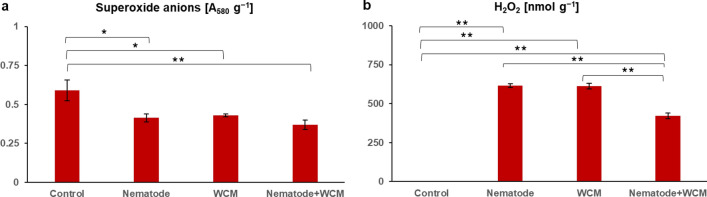
The contents of superoxide anions (**a**) and hydrogen peroxide (H_2_O_2_) (**b**) in the leaves of the spring barley *Hordeum vulgare* plants cultivated for 18 days on commercial horticultural substrate after the cereal cyst nematode *Heterodera filipjevi* and the wheat curl mite (WCM), *Aceria tosichella* inoculations. Results are shown as the means ± SD. Asterisks indicate means which are significantly different at *p < 0.05 and **p < 0.01 according to one-way analysis of variance and a post-hoc Tukey’s test. Control means the nematode-uninoculated and the WCM-uninoculated control plants

The level of superoxide anions was about 0.6-fold lower in N, WCM and N + WCM plants than in C plants (Fig. 1a). The applied analytical method allowed to state that C plants had no H_2_O_2_ in leaves (Fig. 1b). In contrast, plants under three stressful treatments presented the enhanced content of H_2_O_2_. N and WCM plants had its similar level about 615 nmol g^−1^. Significant decrease in H_2_O_2_ content to about 422 nmol g^−1^ was noted in N + WCM plants in relation to N and WCM ones (Fig. 1b).

### Enzymes

**Fig. 2:**
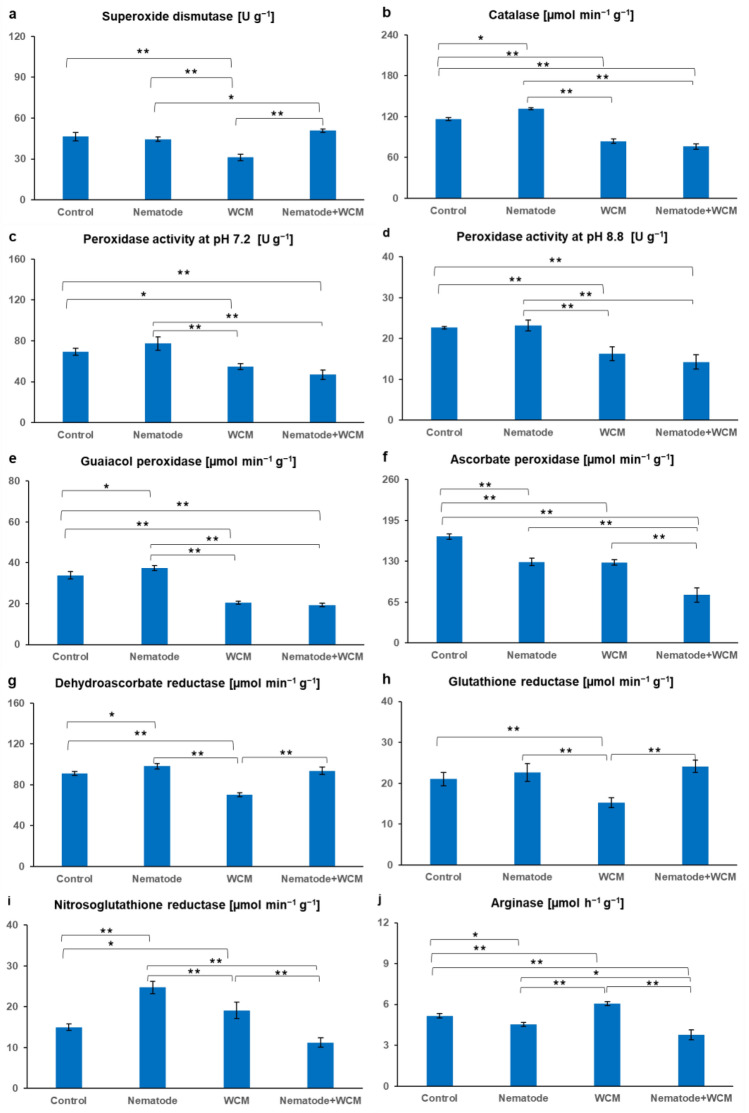
The activity of enzymes (**a**–**j**) in the leaves of the spring barley *Hordeum vulgare* plants cultivated for 18 days on commercial horticultural substrate after the cereal cyst nematode *Heterodera filipjevi* and the wheat curl mite (WCM), *Aceria tosichella* inoculations. Results are shown as the means ± SD. Asterisks indicate means which are significantly different at *p < 0.05 and **p < 0.01 according to one-way analysis of variance and a post-hoc Tukey’s test. Control means the nematode-uninoculated and the WCM-uninoculated control plants

**Fig. 3:**
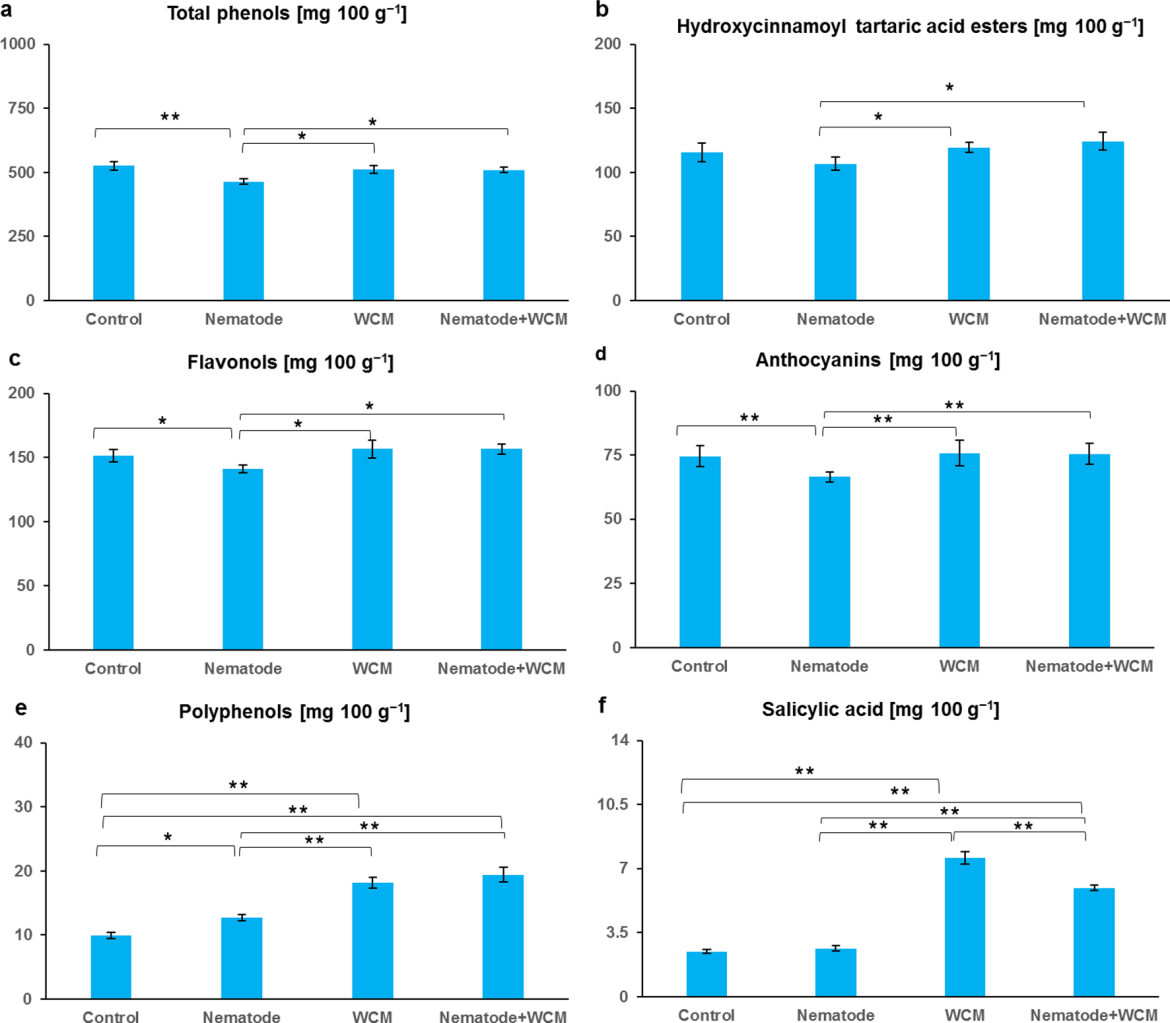
The contents of phenolic metabolites (**a**–**f**) in the leaves of the spring barley *Hordeum vulgare* plants cultivated for 18 days on commercial horticultural substrate after the cereal cyst nematode *Heterodera filipjevi* and the wheat curl mite (WCM), *Aceria tosichella* inoculations. Results are shown as the means ± SD. Asterisks indicate means which are significantly different at *p < 0.05 and **p < 0.01 according to one-way analysis of variance and a post-hoc Tukey’s test. Control means the nematode-uninoculated and the WCM-uninoculated control plants

The activity of SOD was found to be 0.7-fold lower in WCM than in C plants and 1.6-fold higher in N + WCM plants in comparison to WCM plants and 1.1-fold higher in N + WCM plants in comparison to N plants (Fig. 2a). The CAT activity was up-regulated by 1.1-fold in N plants in relation to control plants and down-regulated by about 0.7-fold in WCM and N + WCM plants in relation to C plants. Furthermore, the activity of this enzyme was 0.6-fold lower in N + WCM plants in comparison with C ones (Fig. 2b). The activities of class III peroxidases, including POD at pH 7.2 and 8.8 as well as GOPX, had similar patterns. Their activity decreased about 0.7-fold in WCM and N + WCM plants in relation to C plants, while N + WCM plants presented about 0.6-fold lower activities than N plants. The activity of POD_7.2_ and POD_8.8_ was 30% lower in WCM plants than in N plants and in the case of GOPX by about 50% (Fig. 2c–e). The activity of APX, a member of class I peroxidases, was found to be decreased in N, WCM and N + WCM plants by 0.8-, 0.8- and 0.5-folds, respectively, in comparison to C plants and its activity was significantly diminished by 0.6-fold in N + WCM plants as against N and WCM plants (Fig. 2f). As Fig. 3g presents, the DHAR activity was slightly stimulated (1.1-fold) in N plants in relation to C ones. The WCM plants had the lowest activity of this enzyme, which reached the level around 70 µmol min^−1^ g^−1^. However, the DHAR activity was increased in N + WCM plants (about 25%) in comparison with WCM plants (Fig. 2g). In turn, the activity of GR was similar in C, WCM and N + WCM plants and it was kept at the level of 23 µmol min^−1^ g^−1^ (Fig. 2h). As a result of WCM infestation, the GR activity was significantly down-regulated by 1.6-fold and 0.7-fold in relation to N + WCM and C barley plants, respectively (Fig. 2h). In leaves of barley plants separately infested with CCN or WCM the activity of the GSNOR was increased by 1.6-fold upon CCN and 1.3-fold upon WCM infection in relation to the C plants, whereas in N + WCM plants the 0.6-fold reduction of the GSNOR activity was noted in comparison with both N and WCM plants (Fig. 2i). The highest activity of ARG was observed in WCM plants (about 6 µmol h^−1^ g^−1^) and the lowest (about 4 µmol h^−1^ g^−1^) in N + WCM plants. In the comparison with C plants, the ARG activity of N and N + WCM plants was diminished about 0.9- and 0.7-folds, respectively (Fig. 2j).

### Phenolic metabolites

The parameters including total phenols, hydroxycinnamoyl tartaric acid esters, flavonols, anthocyanins, polyphenols and salicylic acid differed significantly in the analysed four experimental conditions (Fig. 3a–f). The content of total phenols was decreased by about 10% in N plants in relation to the C plants and it increased by about 10% in N + WCM plants in comparison to N ones (Fig. 3a). It was found that the hydroxycinnamoyl tartaric acid ester content was about 10% significantly higher in WCM and about 15% higher in N + WCM plants than in N plants (Fig. 3b). The flavonol content was decreased by about 10% in N plants in relation to the C plants and it was increased by about 10% both in WCM and N + WCM plants in comparison with N plants (Fig. 3c). The anthocyanin content was decreased by about 12% in N plants in relation to the C plants and it was increased by about 12% both in WCM and N + WCM plants in comparison with N plants (Fig. 3d). The content of polyphenols was about 20% higher in N plants and about 50% higher both in WCM and N + WCM plants than in the C plants and it was about 30% higher both in WCM and N + WCM plants than in N plants (Fig. 3e). The level of salicylic acid was significantly enhanced by about 70% in WCM and by about 60% in N + WCM plants in comparison with C plants. Furthermore, N + WCM plants had about 55% more and about 20% less salicylic acid in leaves as against N and WCM ones, respectively (Fig. 3f).

### Lipid peroxidation

**Fig. 4:**
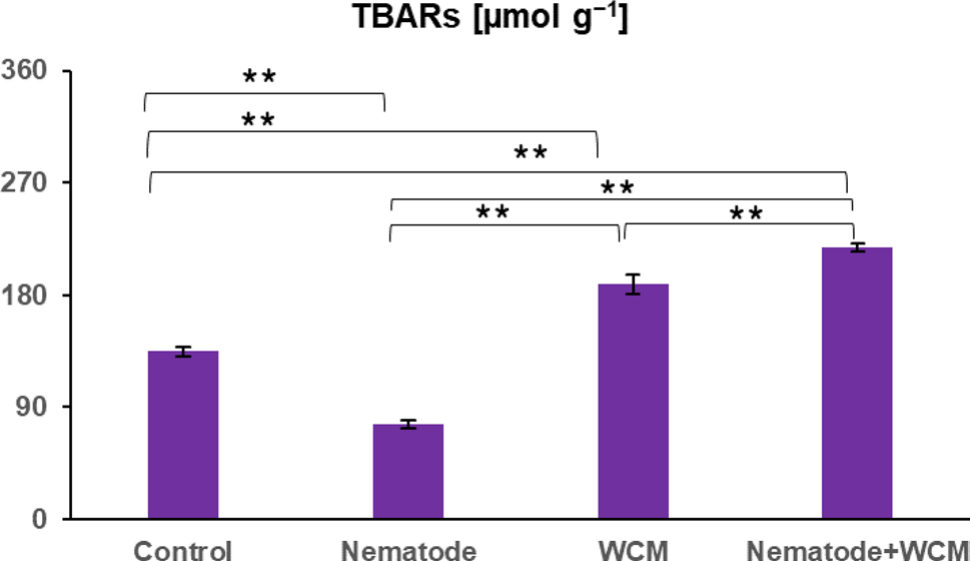
The amount of 2-thiobarbituric acid reactive substances (TBARs) in the leaves of the spring barley *Hordeum vulgare* plants cultivated for eighteen days on commercial horticultural substrate after the cereal cyst nematode *Heterodera filipjevi* and the wheat curl mite (WCM), *Aceria tosichella* inoculations. Results are shown as the means ± SD. Asterisks indicate means which are significantly different at **p < 0.01 according to one-way analysis of variance and a post-hoc Tukey’s test. Control means the nematode-uninoculated and the WCM-uninoculated control plants

The highest content of TBARs was observed in N + WCM plants (about 220 µmol g^−1^) and the lowest (about 75 µmol g^−1^) in N plants. TBAR level was 0.6-fold diminished in N, and it was enhanced by 1.4- and 1.6-folds in WCM and N + WCM plants in comparison with C plants. Moreover, the amount of TBARs was 2.9-fold higher in N + WCM than in N plants and 1.2-fold higher in N + WCM than in WCM plants (Fig. 4).

### Protein carbonylation

**Fig. 5:**
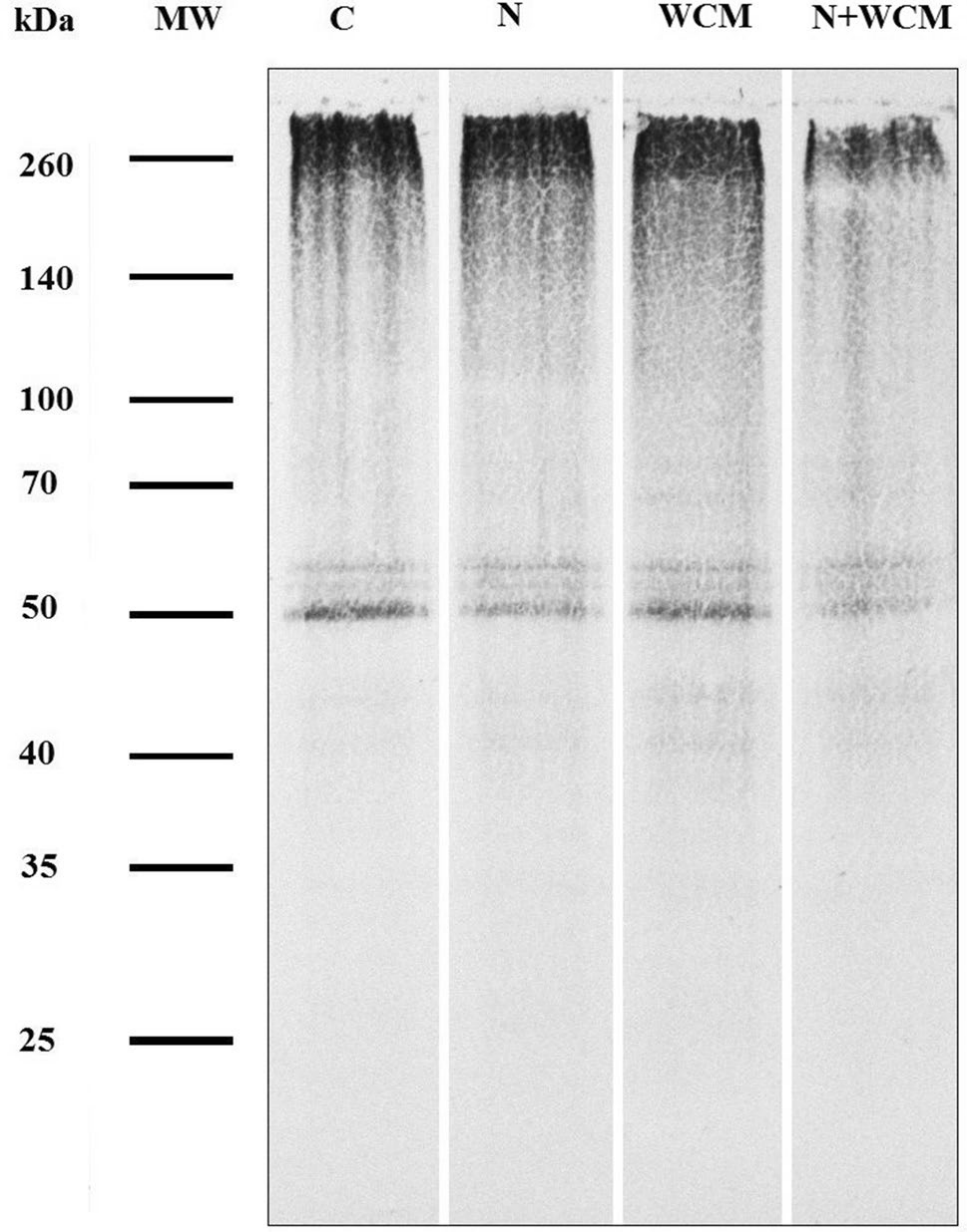
Patterns of carbonylated proteins in the leaves of the spring barley *Hordeum vulgare* plants cultivated for eighteen days on commercial horticultural substrate after the cereal cyst nematode *Heterodera filipjevi* and the wheat curl mite (WCM), *Aceria tosichella* inoculations. Abbreviations in order of their occurrence: *kDa* kiloDaltons, *MW* molecular weight, *C* nematode-uninoculated, *WCM* uninoculated controls, *N* nematode-inoculated, *WCM* WCM-inoculated, *N + WCM* both nematode-inoculated and WCM-inoculated plants

In addition to the TBARs content, the intensity of the protein carbonylation processes, the protein marker of the oxidative damage in plants, was also ascertained (Fig. 5). In response to the separate N and WCM inoculation, the level of protein carbonylation increased in comparison with the C plants. The N inoculation caused the increase in the total protein oxidation level by approximately 12%, and the WCM inoculation by 36% in comparison with C plants. However, the combination of N and WCM led to decrease in the intensity of carbonylation level by 8% against C plants. Moreover, also patterns of oxidized (carbonylated) proteins changed in response to stresses. In the WCM and N + WCM plants carbonylated proteins with molecular weight ~ 72 and 65 kDa were clearly visible, but they were not noticeable in C and N plants. The intensity of bands with MW ~ 55 and 53 kDa was almost constant in all experimental conditions. The level of 50 kDa band was similar in C and WCM plants, but its intensity was lower in N plants, and the lowest in double-stressed barley specimens (N + WCM). Also changes in bands with 45 and 42 kDa differed between experimental variants. Those two bands were the most intense in WCM plants, lower intensity was noticed in N and WCM ones and almost no carbonylation of these proteins was observed in control plants (Fig. 5).

### Photosynthesis

**Fig. 6:**
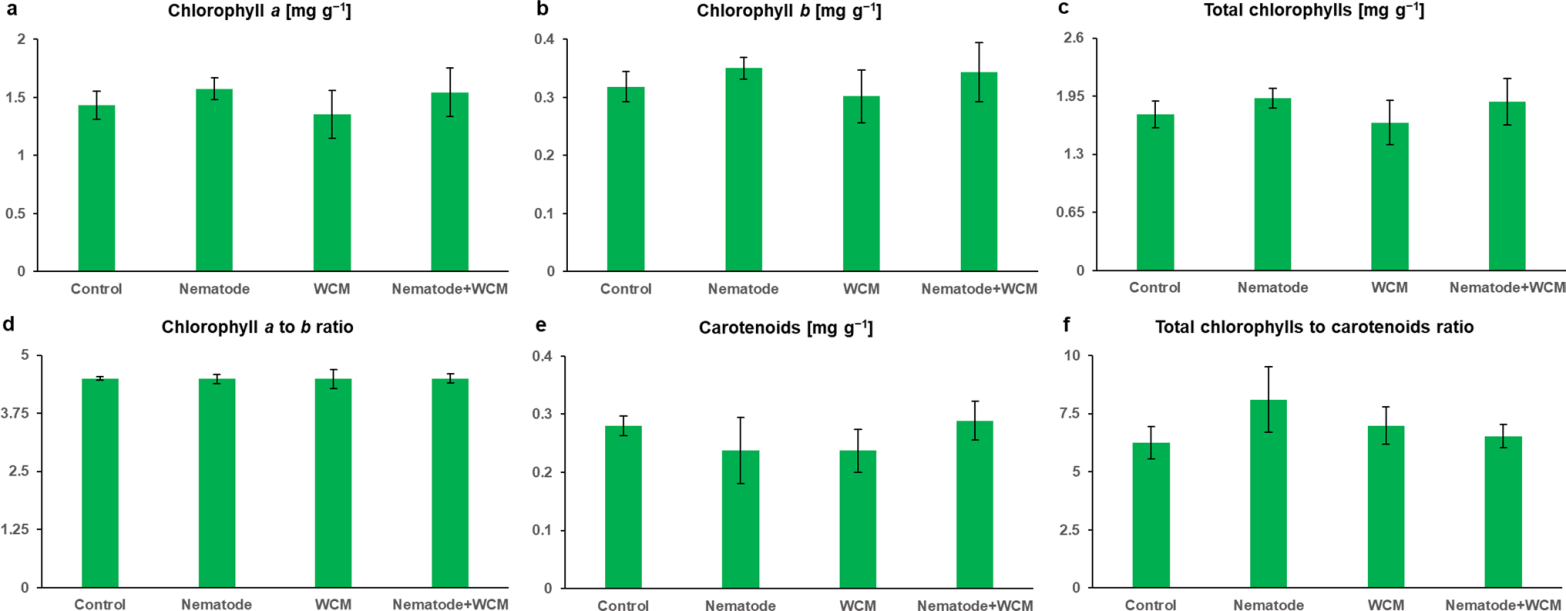
Photosynthetic pigment contents and their ratios (**a**–**f**) in the leaves of the spring barley *Hordeum vulgare* plants cultivated for eighteen days on commercial horticultural substrate after the cereal cyst nematode *Heterodera filipjevi* and the wheat curl mite (WCM), *Aceria tosichella* inoculations. Results are shown as the means ± SD. Control means the nematode-uninoculated and the WCM-uninoculated control plants

**Fig. 7:**
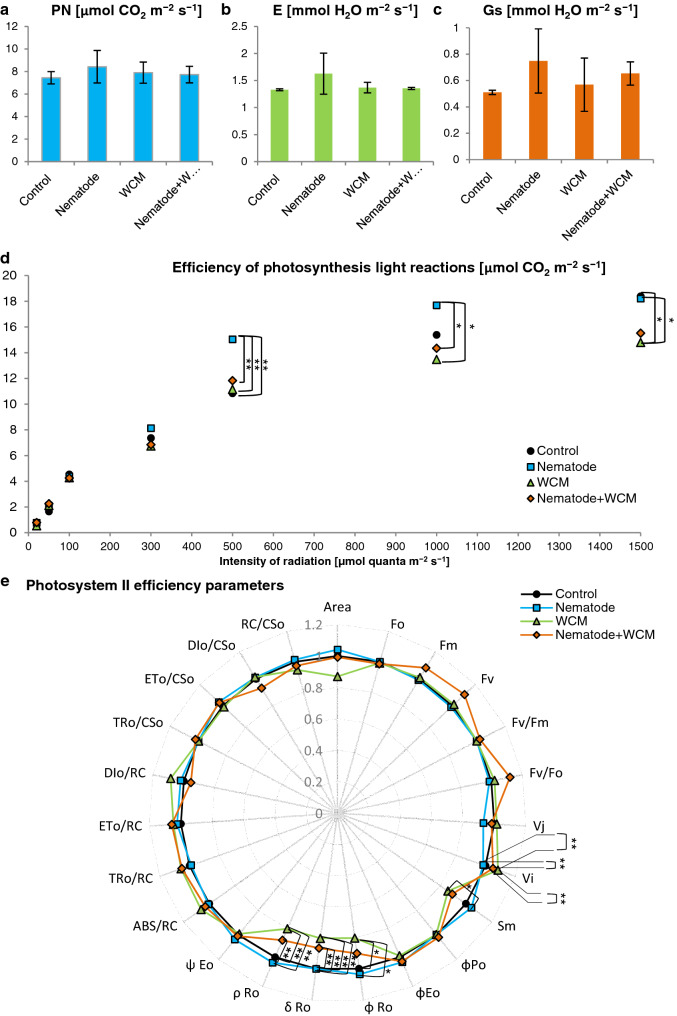
Photosynthesis (**a**–**d**) and photosystem II (PSII) efficiency (**e**) in the leaves of the spring barley *Hordeum vulgare* plants cultivated for eighteen days on commercial horticultural substrate after the cereal cyst nematode *Heterodera filipjevi* and the wheat curl mite (WCM), *Aceria tosichella* inoculations. Results are shown as the means ± SD. Asterisks indicate means which are significantly different at *p < 0.05 and **p < 0.01 according to one-way analysis of variance and a post-hoc Tukey’s test. Control means the nematode-uninoculated and the WCM-uninoculated control plants; *PN* net photosynthesis, *Gs* stomatal conductance, *E* rate of transpiration

The photosynthetic pigment composition in leaves of various experimental plant groups was not significantly differed (Fig. 6). Although differences in photosynthetic pigment contents were not at a statistically significant level, measurements of chl *a* fluorescence provided substantial and interesting results. Actual photosynthesis efficiency did not change statistically between evaluated treatments as evidenced by unchanged net photosynthesis, stomatal conductance and the rate of transpiration (Fig. 7a–c). However, analysis of photosynthesis efficiency at higher radiation intensities revealed statistically significant differences in light reactions efficiency in plants under different treatments (Fig. 7d). Between radiation of 500 and 1500 µmol (quanta) m^−2^ s^−1^, light reactions efficiency was significantly higher in plants inoculated with nematodes than in plants inoculated with mites and plants inoculated with both nematodes and mites. Moreover, at the highest radiation (1500 µmol m^−2^ s^−1^), efficiency of light reactions was significantly lower in WCM plants than in C plants (Fig. 7d). Furthermore, the measurement of chl *a* fluorescence also revealed some changes in PSII efficiency between barley plants uninoculated and inoculated with different pests. Values of PSII parameters, presented on the radar chart, were normalized in relation to a control value (Fig. 7e). The rate of reduction of PSII acceptor side (*V*_*I*_) increased significantly in plants inoculated with WCM in comparison with control plants and nematode-inoculated plants. In turn, parameters related to the electron flows (*ρ*_Ro_, *δ*_Ro_) and quantum yield for the reduction of end acceptors of PSI per photon absorbed (*φ*_Ro_) decreased in WCM plants in comparison with C and N plants (Fig. 7e). Also, total electron carriers per reaction centre (Sm) were reduced in these plants in relation to N-inoculated plants (Fig. 7e). Some changes were also noted in electron flow parameters (*ρ*_Ro_, *δ*_Ro_) of N + WCM plants, which were reduced as compared with N plants (Fig. 7e).

### Chloroplast ultrastructure

**Fig. 8:**
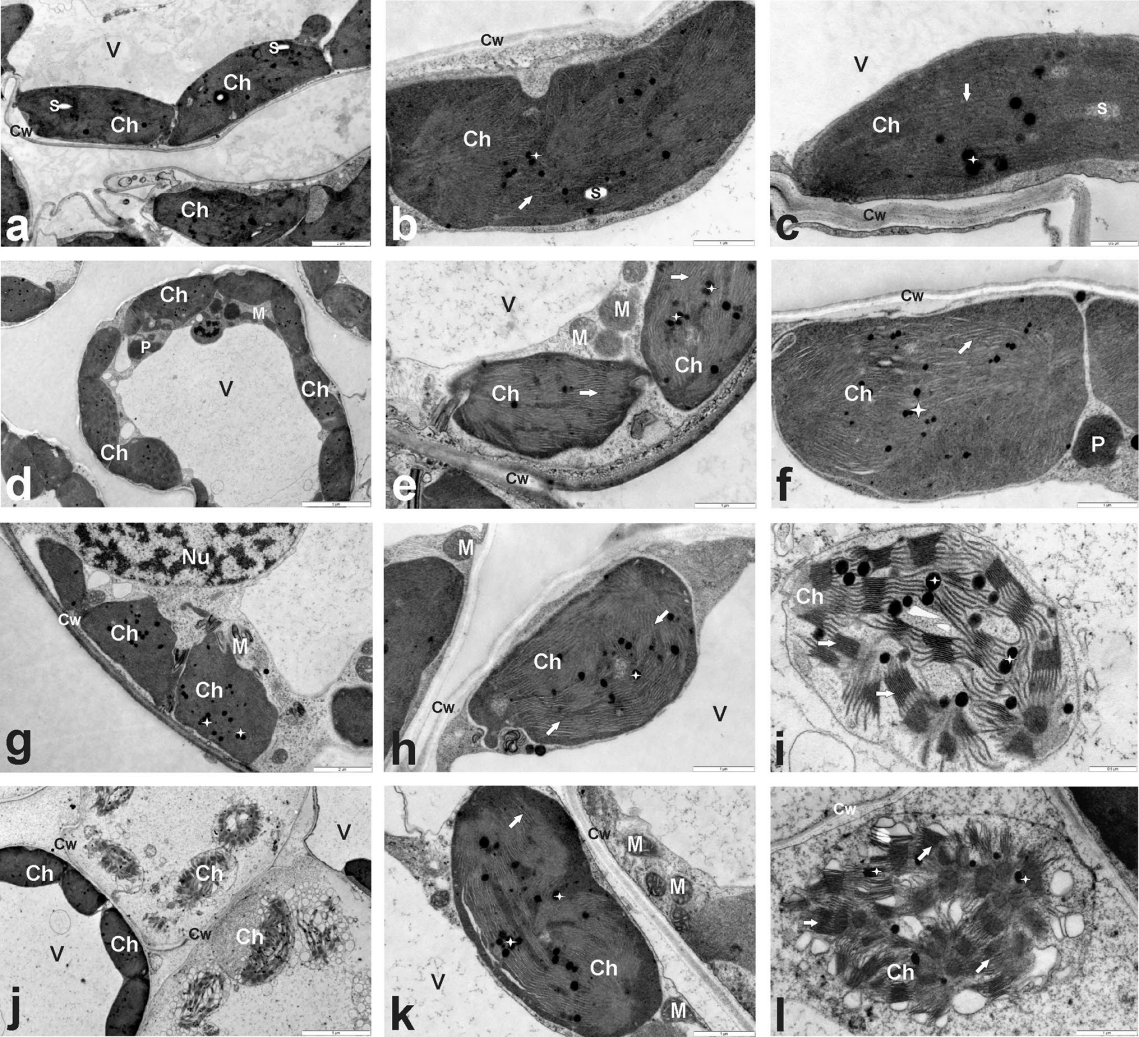
Ultra-thin sections taken from the leaves of the spring barley *Hordeum vulgare* plants cultivated for eighteen days on commercial horticultural substrate after the cereal cyst nematode *Heterodera filipjevi* and the wheat curl mite (WCM) *Aceria tosichella* inoculations. **a**–**c** Nematode-uninoculated and WCM-uninoculated controls, **d**–**f** nematode-inoculated plants, **g**–**i** WCM-inoculated plants, **j**–**l** plants inoculated simultaneously with WCM and *H. filipjevi*. The bar values are shown directly in the figure. Abbreviations: *Ch* chloroplast, *Cw* cell wall, *M* mitochondrion, *Nu* nucleus, *P* peroxisome, *S* starch grain, *V* vacuole, *arrow* thylakoids, *stars* plastoglobuli

As some photosynthesis parameters differed between each experimental group, we used transmission electron microscopy to find out whether these changes go hand in hand with the changed ultrastructure of chloroplasts. Chloroplasts of plants from treatments differed in the shape, the thylakoid structure, the stroma density and the presence of starch grains; however, their distribution and number in leaf cells were like each other (Fig. 8). In the mesophyll of control plants, chloroplasts were ellipsoidal in shape and they had a regular structure of thylakoids,  electrondense stroma and numerous plastoglobules. Additionally, some small starch grains were occasionally observed (Fig. 8a–c). In leaves of N- (Fig. 8d–f) or WCM-infected plants (Fig. 8g–i), irregularly shaped chloroplasts with swollen (Fig. 8d–h) or  electrontranslucent (Fig. 8i) stroma were found. Apart from these malformations, chloroplasts of infested plants had rather regular arrangement of thylakoid membranes with well-developed grana. In turn, mesophyll cells of leaves from plants infected simultaneously with nematodes and mites contained chloroplasts with different degrees of degradations (Fig. 8j–l). In these chloroplasts, the stroma was electrontranslucent, whereas the thylakoid system was well visible. Interestingly, besides properly developed cells with chloroplasts similar to control ones, cells with far-reaching changes were found, probably in these leaf part where *A. tosichella* fed (Fig. 8j, k). In such degraded chloroplasts the dilated thylakoids forming many vesicles occurred apart from typically arranged ones (Fig. 8l). What is more, no starch grains were noted in all chloroplasts of infested plants, but numerous plastoglobuli were still observed.

## Discussion

As sessile organisms, plants are equipped with sophisticated defence apparatus involving non-enzymatic and enzymatic antioxidants to cope with oxidative stress and to promote photosynthesis efficiency. Regulation of ROS and photosynthetic metabolism underlies plant successful responses against numerous environmental stressors (Suzuki et al. 2014; Choudhury et al. 2017; Woźniak et al. 2019; Morales et al. 2020).

The photosynthetic apparatus is one of the most sensitive sensors of redox changes in the plant, which allows it for effective acclimatization to altering environmental conditions (Goltsev et al. 2016). Biotic and abiotic stress factors cause redox imbalance in the plant, leading to a reduction of photosynthesis efficiency, an activation of alternative metabolic pathways and a synthesis of secondary metabolites (Piwowarczyk et al. 2018; Tokarz et al. 2018, 2020b; Makowski et al. 2019; Rozpądek et al. 2019). Attack and colonization of plant by pests lead to an increased demand for primary metabolites, mainly carbohydrates, and imply the activation of a defence response requiring an increased production of secondary metabolites (Blasi et al. 2015). Feeding mites secrete into the leaves specific mix consisting of elicitors and fatty and amino acids, which on the one hand enable the plant to recognize the pest, but on the other hand, lead to host tissue and cell damage (Gilardoni et al. 2010). As a result, an intensive production of ROS occurs leading to membrane lipid peroxidation, DNA degradation, redox and photosynthesis efficiency disorders (Blasi et al. 2015). The reduction of photosynthesis may be induced by either direct mesophyll damage and a significant reduction in stomatal conductance (Fadini et al. 2004) or disorders in the transcription and translation of proteins associated with the photosynthetic apparatus (Schmitt et al. 2014) or disruptions in the synthesis pathway of chlorophylls and carotenoids (Bronner et al. 1991). It was shown that reduced photosynthesis efficiency in rice plants can be caused not only by a decrease in chlorophyll content (Buffon et al. 2016, 2018) but also by down-regulation of seven proteins synthesis related to NADPH production and thus to adenosine triphosphate and glucose synthesis (Blasi et al. 2017). In the presented experiment, however, no significant differences were observed in the efficiency of photosynthesis (PN), transpiration (E), as well as in stomatal conductance (Gs) between barley plants inoculated and uninoculated with CCN and WCM. In addition, no differences were found in the content of photosynthetic pigments between these plants. However, the photosynthetic light response curve revealed that the presence of mites (apart from or with nematodes) significantly reduced the efficiency of CO_2_ assimilation what some malformations in the chloroplast ultrastructure accompanied simultaneously. At the same time, inoculation with nematodes increased photosynthesis efficiency. Therefore, to determine the real condition and efficiency of photosynthetic apparatus, the OJIP fast-fluorescence test based on non-invasive chl *a* fluorescence measurement was applied. Stress factors leading to redox imbalance disturb both photochemical and biochemical phases of photosynthesis (Tokarz et al. 2020b). Stressors reduce the number of electrons reaching the PSII reaction center (RC) because of disturbances in the oxygen-evolving complex (OEC) (Nikkanen et al. 2017). This PSII donor side limitation threatens with the appearance of the strongest biological oxidant the excited primary electron donor of PSII (P680*). On the other hand, redox homeostasis disorders on the PSII acceptor side lead to a reduction in the rate and efficiency of electron transport between PSII and PSI and thus to PSI oxidation (Tokarz et al. 2020a). In contrast, the limitation on the PSI acceptor side involves PSI over-reduction (Tokarz et al. 2020a). All these limitations lead to generation of ROS, RNS and organic radicals causing irreversible destruction of the photosynthetic apparatus (Kalaji et al. 2016; Tokarz et al. 2020a). Kinetic of chl *a* fluorescence revealed that rice plants sensitive to mite feeding had a significantly lower number of open PSII RC (F_0_) among all PSII RC (Vj) as well as significantly lower efficiency of trapped energy flux (TRo/CSo) compared to resistant plants (Buffon et al. 2018). At the same time, sensitive cultivars dissipated more intensely part of the trapped energy (Dio/RC) (Buffon et al. 2018). Barley plants examined in this work, regardless of kind of pest inoculation, were characterized by the same efficiency of trapping and transporting radiation to PSII RC as control plants. At the same time, no limitations on the PSII donor side were observed indicating no damage of OEC. On the other hand, inoculation with WCM and N + WCM caused a significant limitation in the efficiency of electron transport outside PSII RC. Limitation resulted from decreased pool of rapidly reducing plastoquinone PQ (*V*_*i*_) as well as the significantly diminished total electron carriers (Sm). Inoculation with WCM, apart from and together with nematode, significantly limited the electron transport on the PSI acceptor side (*ρ*_Ro_, *δ*_Ro_, *φ*_Ro_); thus ROS appeared in barley cells because of these limitations.

We have previously presented enhanced superoxide anion and H_2_O_2_ production in aerial parts of *Arabidopsis thaliana* plants infested with *Heterodera schachtii* (the beet cyst nematode) (Labudda et al. 2018). What is more, Khanna et al. (2019) proved increased generation of superoxide anions and H_2_O_2_ in shoots of tomato plants infected with *Meloidogyne incognita* (root-knot nematode) and Javadi Khederi et al. (2018) documented enhanced level of H_2_O_2_ in *Vitis vinifera* leaves infested with *Colomerus vitis* (the grape erineum mite). In our investigations discussed here, we noted a considerable decrease in superoxide anion accumulation in barley leaves because of separate CCN or WCM infection and of a double infection with CCN and WCM. Diminished superoxide anion content partly arose from superoxide anions’ dismutation into O_2_ and H_2_O_2_ by the activity of SOD. However, since the SOD activity was not very strongly stimulated in stressful conditions, it can be argued that reduced amounts of superoxides were results of another metabolic event. NADPH oxidase (Nox) is an enzymatic source of superoxide anions in plants. Nisimoto et al. (2018) presented that one of the Nox, Nox4 is a hydrogen peroxide sensor and its dehydrogenase domain reacts quickly to H_2_O_2_ in cytosol to modulate the activity of Nox4. Thus, reduced content of superoxide anions in barley plants under three stressful conditions may also result from Nox down-regulation through exaggerated accumulation of H_2_O_2_. As already mentioned, increased content of H_2_O_2_ was noted in leaves of N and WCM inoculated plants. An especially noteworthy finding pertains to approximately 0.7-fold diminished level of H_2_O_2_ in N + WCM plants in comparison with N and WCM plants. This serendipitous result uncovers efficient antioxidative response in double-infested barley, notwithstanding it could be presumed at first glance that barley under biotic stress combination should have this response dysregulated. As it has been formerly postulated (Suzuki et al. 2014; Choudhury et al. 2017), a detoxification of ROS presents a unique status under double stress, what is different than the plant defence reaction to one stress taking place independently. Consequently, the combination of two biotic stressors (cyst nematode incepting nutrients from roots and eriophyoid mite from leaves) imposed on spring barley plants a special physiological acclimatization to two biotic stress factors.

Plants protect themselves against oxidative stress consequences induced by pests and pathogens among others through antioxidative enzymes. Class III peroxidases (POD and GOPX) and CAT had the same activity patterns in plants under three stress situations as well as in control plants. The activity of GOPX and CAT was significantly stimulated in N-infested plants in comparison with control ones. Similar results were observed earlier in *A. thaliana* and *Lycopersicon esculentum* plants infected with nematodes (Labudda et al. 2018; Khanna et al. 2019). In the present results, elevated activities of GOPX and CAT were not reflected in the reduced content of H_2_O_2_. This can suggest that the H_2_O_2_ production was greater than the ability to scavenge H_2_O_2_ by GOPX and CAT. The increased activities of GOPX and CAT corresponded to a reduced content of phenols, including flavonols. The peroxidase-flavonoid mechanism of the ROS deactivation often occurs in plant cells, and during this GOPX degrades phenolic molecules with simultaneous neutralisation of H_2_O_2_ (Takahama and Oniki 2000). Furthermore, CAT can act as a bifunctional enzyme; on the one hand, CAT catalyses decomposition of H_2_O_2_, but on the other hand it can oxidize hydrogen donors such as phenolic molecules with consumption of peroxides (Chen et al. 2017). Therefore, it can be assumed that both above described systems (peroxidase-flavonoids and catalase-phenols) participated in controlling H_2_O_2_ level in leaves of nematode-infected barley. Interestingly, the postulated catalase-phenol activity corresponded well to the lower lipid peroxidation in nematode-infected plants that might partly be due to consumption of lipid peroxides by CAT. As described above, plants have to counteract tear ROS homeostasis down during stress, but phytophagous mites seek to colonise effectively of plant hosts among others owing to secreting salivary protein effectors into leaves to modulate/curtail plant defence mechanisms (Jonckheere et al. 2016). Attention is drawn to the inhibited activity of PODs and GPOX in WCM and N + WCM plants in this context. Hemetsberger et al. (2012) showed that *Ustilago maydis* secreted effector Pep1 (Protein essential during penetration-1) into the maize leaves to inhibit of host peroxidase activity and to establish of a biotrophic interaction. Although this type of effector has not yet been identified in mites, our results may suggest that WCM deliberately modulated the peroxidase activity to prevent the activation of class III peroxidase-dependent defence responses such as lignin and suberin production, the cell wall components cross-linking or formation of phytoalexins (Almagro et al. 2009; Minibayeva et al. 2015).

APX, belonging to class 1 peroxidases, takes part in H_2_O_2_ neutralisation by Foyer–Halliwell–Asada pathway consisting of GSH and ASA as a key non-enzymatic antioxidant compounds and enzymes such as DHAR, GR and monodehydroascorbate reductase (Labudda 2018). Our results indicate that the decomposition of H_2_O_2_ in the leaves of C plants depended on APX activity, because its stimulated activity was noted. However, regarding our main research question (how does barley react to the double infestation?), our finding that the APX activity was diminished in N + WCM plants in relation to N and WCM plants indicates that H_2_O_2_ scavenging in N + WCM plants was re-routed to non-enzymatic H_2_O_2_ decomposition during direct reaction with ASA. Decreased APX activity ensured ASA molecules that reacted with H_2_O_2_ and scavenged them (Grinstead 1960). The product of the H_2_O_2_ reaction with ASA was its oxidized form, DHA, which could be a substrate for DHAR. This antioxidant mechanism from Foyer–Halliwell–Asada pathway was in operation efficiently because the activity of DHAR in N + WCM plants was on the same level than in C plants; hence the recovery of ASA by DHAR still occurred. One more discovery supports our interpretation. DHAR tapped two molecules of GSH to produce one molecule of ASA but GSSG was equally formed. The increase of GR activity in N + WCM plants was noted. Since GR transforms GSSG to GSH, regenerated GSH pool was constantly achievable and/or GSH could anew begin reaction of DHAR. GSH could be also oxidized to GSSG by the direct reaction with H_2_O_2_, which in this way was beneficially removed from cells (Abedinzadeh et al. 1989; Labudda 2018; Ding et al. 2020). Summarizing, observed by us, decreased number of H_2_O_2_ in N + WCM plants was presumably due in no small part to the Foyer–Halliwell–Asada pathway. In addition, this mechanism could be supported by polyphenols, which through reaction with ROS/RNS restricted oxidative damage in stressed barley plants (Hussain et al. 2016).

Reactive nitrogen species (RNS), including nitric oxide (NO) that is in the leading position amid RNS, are important regulatory molecules during undisturbed plant ontogenesis. They also participate in the cellular signalling under stressful conditions (Corpas 2017). Moreover, NO can reversibly bind the sulfhydryl groups of cysteines, so *S*-nitrosothiols (SNOs) are produced, including GSNO, a biochemical stock of NO in cells (Jahnová et al. 2019). Metabolism of GSNO in cells is controlled by GSNOR, catalysing reduction (reliant on NADH) of GSNO to GSSG and ammonia (NH_3_) (Corpas and Barroso 2013). Kovacs et al. (2016) and Begara-Morales et al. (2019) presented claims that ROS can inhibit the activity of GSNOR resulting in the accumulation of GSNO and significantly increased H_2_O_2_ neutralisation through the Foyer–Halliwell–Asada pathway. Other research showed GSNO-dependent irreversible inhibition of the enzyme activity of APX (Clark et al. 2000). This APX inhibition by GSNO strengthens our explanation regarding down-regulated activity of APX in N + WCM plants. Our results are in accordance with Kovacs et al. (2016) and Begara-Morales et al. (2019) and indicate that antioxidant capacity of N + WCM barley plants to some extent came from the inhibition of GSNOR and the accumulation of GSNO. Moreover, Jahnová et al. (2019) proposed that diminished GSNOR activity with simultaneous increase in the GSNO level can lead to improved plant resistance to pest/pathogen infections. Regardless of this, the up-regulated GSNOR activity in N and WCM plants was documented, thus scavenging the intolerable amount of NO through the activity of GSNOR took place. Therefore, barley plants under separately CCN and WCM infestation were protected from effects of the high concentrations of RNS that are toxic for plants (Corpas and Barroso 2013).

Our earlier published article indicated that alterations in nitrogen metabolism occurred in plants infected with the beet cyst nematode (Labudda et al. 2020a). Arginase (ARG) is not an enzyme that has direct influence on ROS deactivation, but it provides ornithine for the synthesis, e.g. polyamines, important metabolites during plant diseases (Walters 2003). Ornithine decarboxylase initiates the ARG-dependent polyamine synthesis pathway, so ornithine is decarboxylated to putrescine. From putrescine spermidine synthase produces spermidine, and spermine synthase synthesizes spermine from spermidine. Putrescine, spermidine and spermine are positively charged metabolites; therefore, they can bind to negatively charged compounds and neutralize ROS (Hasanuzzaman et al. 2019). It cannot be excluded that enhanced activity of ARG in WCM plants can promote synthesis of polyamines and indirectly improve non-enzymatic antioxidant apparatus in these plants. However, another mechanism is also possible. The enhanced ARG activity, and thus possible stimulated polyamine synthesis, in combination with the observed accumulation of salicylic acid, a master mediator of plant defence (Palmer et al. 2017), may point to defence strategy induced by barley plants to prevent excessive development of WCM populations on infested plants. In reaction to devouring pests, attacked plants synthesize and emit herbivore-induced plant volatiles ‘as a call for help’ to attract carnivorous natural enemies of herbivores. Due to these volatiles, plants can indirectly decrease the number of herbivores by more than 90% (Kessler and Baldwin 2001). The exogenous treatment with putrescine, spermidine and spermine led to emission of volatiles in *Phaseolus lunatus* leaves, among others methyl salicylate, and its content was particularly high after treatment with putrescine (Ozawa et al. 2009). These authors also presented that *P. lunatus* leaves treated with spermine + jasmonic acid at the same time attracted more predatory mites *Phytoseiulus persimilis* than those treated with jasmonic acid singly. It has been suggested that spermine has an important role in the regulation of synthesis of volatiles induced by *Tetranychus urticae* (Ozawa et al. 2009). Furthermore, Shimoda et al. (2002) proved that two predators of *T. urticae*, thysanopteran *Scolothrips takahashii* and coleopteran *Oligota kashmirica benefica* exhibited preference for methyl salicylate or methyl salicylate + jasmonic acid-treated *P. lunatus* leaves when compared with *T. urticae*-uninfested leaves. Taken together, our results can suggest that stimulated polyamine synthesis through ARG reaction and co-existing enhanced level of free and conjugated forms of salicylic acid in WCM-infected barley plants can indicate that barley plants try to cope with herbivores through increased synthesis of volatiles to lure carnivorous insects. Such defence response of barley plants has been shown in article by Ninkovic et al. (2001). These authors proved that volatiles synthetized by the bird cherry-oat aphid (*Rhopalosiphum padi*)-infested barley plants attracted adults of the seven-spotted ladybird (*Coccinella septempunctata*). Ninkovic et al. (2001) concluded that aphid-induced barley volatiles had an important role in food-searching behaviour of ladybirds and this behaviour can be inborn or effect of associative learning of *C. septempunctata* imagines.

In our research, we implemented two biochemical markers for the assessment of oxidative damage in barley cells. We began with scrutiny of TBARs level for the detection of lipid oxidation products, such as 4-hydroxy-2-nonenal and malondialdehyde and other toxic aldehydes, alkenals and hydroxyalkenals (Alché 2019). The observed lowest level of TBARs in N plants (even lower than in C plants) indicates that the antioxidative mechanisms were efficient enough to limit the oxidative damage of cell membranes, as we have previously suggested to some extent this may be due to catalase activity against peroxides through the pathway catalase-phenols (Chen et al. 2017). A high level of TBARs was observed in barley plants on whose leaves WCM individuals were feeding. We suppose it could have a double effect on physiological processes in these plants. On the one hand, negative because the integrity of some cell membranes of WCM and N + WCM plants was destroyed what corresponded with different degrees of degradations of chloroplasts in these plants. However, the positive side of this state is also possible, with a direct impact on increased barley defence mechanisms against WCM. First, the increased accumulation of lipid oxidation products in WCM-infested leaves makes them a less favourable nutrient source for WCM. A similar observation was made by Shukle and Murdock (1983). It was observed that the plant diets containing the soybean prooxidant enzyme lipoxygenase (LOX) (initiating various oxygenated compounds production) led to retarded *Manduca sexta* larval growth, the insect pest of plants from the family Solanaceae. Second, wounds induced by WCM feeding on barley leaves could stimulate releasing of fatty acids from membrane lipids such as linolenic acid and next LOX could convert linolenic acid to jasmonic acid and further volatile aldehydes can be produced (Mosblech et al. 2009; Rahimi et al. 2016). This would be consistent with the above postulated role of arginase in the production of polyamines and stimulated by them the emission of volatiles. Moreover, Hildebrand et al. (1986) presented that because of *T. urticae* infestation on soybean leaves, a large increase in TBAR content and a significant enhancement in LOX activity co-occurred.

The second marker of oxidative stress was the carbonylation of proteins expressed in amount of the C = O groups incorporated into proteins because of predominantly lysine arginine, proline and threonine residues oxidation (Kalemba and Pukacka 2014). The above presented antioxidant mechanisms operating together indicated efficient antioxidant capacity of N + WCM plants because the combination of N and WCM stresses led to the lowest intensity of carbonylation level against other plant groups. Therefore, it can be assumed that wastage of protein functions was significantly diminished. However, upon separate nematode or mite inoculation, the number of C = O groups in proteins was enhanced in comparison with the control plants. Our results are consistent with the research presented by Dworak et al. (2016), who showed that the protein carbonylation level was increased in leaves of *Zea mays* plants infested with *T. urticae* or under water deficit in comparison with control plants. However, the combination of *T. urticae* and water deficit resulted in significantly decreased amount of C = O groups in proteins in comparison to singly *T. urticae* or water deficit-stressed plants as well as control ones. Our results and Dworak et al. (2016) can indicate that the double stress, regardless of whether these are two biotic stresses or a combination of abiotic and biotic stresses, plants react in a similar way. In the future, it would be interesting to examine whether such plant response takes place also in other experimental systems and whether it goes beyond species from the Poaceae family. This discovery can set new research paths because issues related to posttranslational modification of proteins during stress combination are virtually unexplained.

**Fig. 9:**
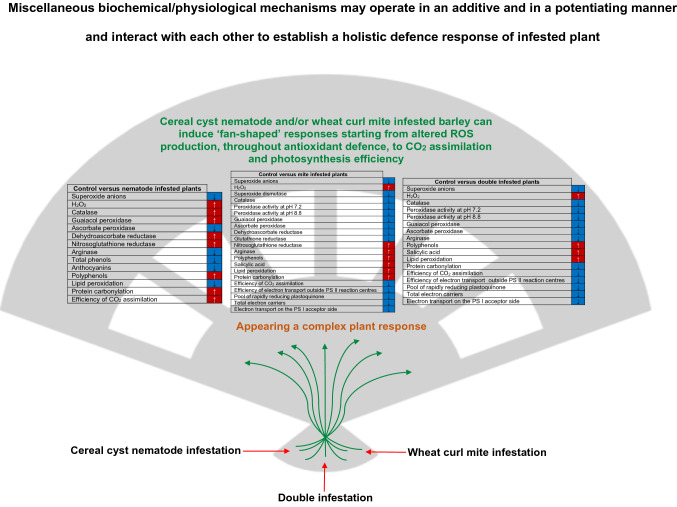
Scheme presenting the postulated ‘fan-shaped’ responses in the leaves of the spring barley *Hordeum vulgare* plants infested with the cereal cyst nematode and the wheat curl mite. All parameters are presented in relation to the control uninfested plants. The upward arrows and the red background indicate an increase, while the downward arrows and the blue background indicate a decrease in the values of the tested parameters. Abbreviations: *PS* photosystem, *ROS* reactive oxygen species

To conclude, the obtained results increase the knowledge of redox metabolism and photosynthesis of cereal plants infested simultaneously with two pests. We unravelled for the first time how barley reacts to stress arisen by cereal cyst nematode and wheat curl mite infestation. It was found that the ROS production and oxidative damage were increased in nematode and mite-infested leaves and prominent enzymatic and non‐enzymatic antioxidants were activated. Furthermore, infestation with mites (apart from or with nematodes) significantly decreased the efficiency of CO_2_ assimilation by leaves of barley plants, but infection only with nematodes increased photosynthesis efficiency. Our investigations point out tight relatedness between the ROS metabolism and the regulation of photosynthesis in leaves of barley plants colonised by nematodes and mites. Summarizing, to manage the stress induced by pest infestation, barley plants can induce a multi-component model of stress response in the form of biochemical-physiological ‘fan-shaped’ defence response (Fig. 9).

## Abbreviations

APX: Ascorbate peroxidase
ARG: Arginase
ASA: Reduced ascorbate
CAT: Catalase
Chl a: Chlorophyll *a*
Chl b: Chlorophyll *b*
DHAR: Dehydroascorbate reductase
FW: Fresh weight
GOPX: Guaiacol peroxidase
GR: Glutathione reductase
GSH: Reduced glutathione
GSNO: *S*-Nitrosoglutathione
GSNOR: Nitrosoglutathione reductase
GSSG: Oxidized glutathione
POD: Peroxidase
PSI: Photosystem I
PSII: Photosystem II
CCN: Cereal cyst nematode
RNS: Reactive nitrogen species
ROS: Reactive oxygen species
SA: Salicylic acid
SNO: *S*-Nitrosothiol
SOD: Superoxide dismutase
TBAR: 2-Thiobarbituric acid reactive substance
WCM: Wheat curl mite
